# Activation of M1 Macrophages in Response to Recombinant TB Vaccines With Enhanced Antimycobacterial Activity

**DOI:** 10.3389/fimmu.2020.01298

**Published:** 2020-06-23

**Authors:** Shiu-Ju Yang, Yih-Yuan Chen, Chih-Hao Hsu, Chia-Wei Hsu, Chun-Yu Chang, Jia-Ru Chang, Horng-Yunn Dou

**Affiliations:** ^1^National Institute of Infectious Diseases and Vaccinology, National Health Research Institutes, Zhunan, Taiwan; ^2^Department of Biochemical Science and Technology, National Chiayi University, Chia-Yi, Taiwan

**Keywords:** recombinant Bacille Calmette–Guérin, *Mycobacterium tuberculosis*, innate immunity, macrophage, vaccine

## Abstract

Pulmonary tuberculosis (TB) is a difficult-to-eliminate disease. Although the Bacille Calmette–Guérin (BCG) vaccine against *Mycobacterium tuberculosis* (MTB) has been available for decades, its efficacy is variable and has lessened over time. Furthermore, the BCG vaccine no longer protects against newly emerged Beijing strains which are responsible for many current infections in adults. Development of a novel vaccine is urgently needed. In this study, we first tested the efficacy of our recombinant BCG vaccines rBCG1 and rBCG2, compared to parental BCG, against MTB strain H37Ra in mice. Both the bacterial load and the level of lymphocyte infiltration decreased dramatically in the three groups treated with vaccine, especially rBCG1 and rBCG2. Furthermore, the Th1 and Th17 responses increased and macrophage numbers rose in the vaccination groups. Th1-mediated production of cytokines TNF-α, IFN-γ, and MCP-1 as well as M1-polarized cells all increased in lung tissue of the rBCG1 and rBCG2 groups. Clodronate-induced depletion of macrophages reduced the level of protection. Based on these results, we conclude that rBCG vaccines induce a significant increase in the number of M1 macrophages, which augments their potential as TB vaccine candidates.

## Introduction

Tuberculosis is a difficult-to-treat disease that causes millions of deaths worldwide (1, 2). The disease is a major public health problem in many countries, consuming considerable societal and financial resources. The bacille Calmette–Guérin (BCG) vaccine, created in 1921, is the only licensed vaccine against *Mycobacterium tuberculosi*s (MTB) (3). When administered during childhood, the vaccine provides ~10–15 years of protection, but this immunity wanes in adulthood (4). However, the protective efficacy of the BCG vaccine varies widely, from 0 to 88% (5–7). Based on this epidemiological evidence, BCG appears to lose its protective effect over time (4–7). Although most studies did not find any association, a study that subdivided Beijing MTB strains into typical and atypical lineages found typical Beijing strains to be more frequent among BCG-vaccinated persons compared to non-vaccinated persons (8), suggesting that BCG vaccination is less protective against typical Beijing strains, which has also been reported in animal models (9, 10). For these reasons, development of a novel, effective vaccine is urgently needed.

Antigen 85B (Ag85B) and culture filtrate protein 10 (CFP-10) are two candidates for effective MTB vaccine development. Ag85B, part of the Ag85 complex with antigens B and C, functions as a mycolyl transferase in MTB cell wall assembly (11). This major protective protein is recognized by T cells and is a strong MTB antigen (12, 13). Ag85B binds fibronectin, which then stimulates human monocytes to produce TNF-α (14). Overexpression of Ag85B reduces bacterial load (15) and protects mice against MTB infection (16). Recently, Prendergast et al. showed that Ag85B-deficient BCG had reduced capacity to infect macrophages, whereas an Ag85B-overexpressing BCG strain had improved uptake by macrophages (17). CFP-10, a secreted protein encoded by the RD-1 region of MTB, combines with the 6-kDa early secreted antigen target 6 (ESAT-6) protein to form a heterodimer (18). Loss of both Ag85B and CFP-10 has been associated with attenuated MTB infection (19, 20). Therefore, these targets have been used as trial vaccine candidates for years. However, some studies show that Ag85B and CFP-10 do not provide adequate protection (17) or do not have enough long-term data to support their efficacy (21, 22).

Recombinant BCG vaccines can be engineered to express immunodominant antigens such as Ag85 complex (Ag85A-C) and ESAT-6. Notably, CD8+ T cells recognizing CFP-10 are elicited following MTB infection in humans (23–26). Therefore, our strategy is to combine Ag85B and CFP-10 into a fusion protein that is expressed in BCG hosts. Human IL-12 provides a robust immune response to such fusion proteins and could enhance vaccine efficacy. Recently, our recombinant BCG vaccines rBCG1 and rBCG2 demonstrated higher stimulation of IFN-γ secretion compared to parental BCG vaccine (27). rBCG1 is BCG containing a fusion protein of Ag85B and CFP-10, whereas rBCG2 is BCG containing the same fusion protein plus human IL-12 (27). Prior to the present study, we had not tested the protective efficacy of these two recombinant vaccines in an animal infection model. In order to establish the efficacy of our rBCG vaccines in mice, we tested them against MTB strain H37Ra administered intravenously and compared their effects to that of the parental BCG vaccine; in this trial, the mice were given two vaccinations 2 weeks apart and then challenged with H37Ra 2 weeks later. This study was designed to evaluate rBCG1 and rBCG2 efficacy and long-term protection against MTB compared to the parental BCG vaccine. The study included characterization of immune cell responses, including cytokine-release profiles.

## Materials and Methods

### Bacterial Strains and Cultures

Parental BCG is *Mycobacterium bovis* BCG (Tokyo172) and was used to construct the recombinant BCG strains rBCG1 and rBCG2 in a previous study (27). Briefly, rBCG1 was constructed using MTB antigens Ag85B and CFP-10 in an *M. bovis* BCG Tokyo 172 strain, and rBCG2 was constructed using MTB antigens Ag85B and CFP-10 plus human IL-12 also in *M. bovis* BCG Tokyo 172. rBCG1 and rBCG2 were compared to *M. tuberculosis* strain H37Ra. H37Ra and BCG (Tokyo 172) were maintained on Middlebrook 7H9 medium (Difco Laboratories, Detroit, MI, USA) and supplemented with 0.5% glycerol, 0.05% Tween 80, and 10% albumin- dextrose-catalase or on solid Middlebrook 7H11 medium (Difco Laboratories) supplemented with oleic acid-albumin-dextrose-catalase. rBCG1 and rBCG2 were maintained on the media described above with 25 μg/ml kanamycin.

### Mice and Immunization

Female C57BL/6 and SCID mice aged 6–8 weeks were purchased from the National Laboratory Animal Center (Taipei, Taiwan). All mice were kept in individually ventilated cage environments at the Animal Center of the National Health Research Institutes (Maoli, Taiwan). The temperature was maintained at 20–24°C with a relative humidity of 40–70% and a 12-h light/dark cycle. Animal experiments were reviewed and approved by the National Health Research Institutes Institutional Animal Care and Use Committee (NHRI-IACUC). We conducted experiments according to guidelines set out by the Association for Assessment and Accreditation of Laboratory Animal Care International (AAALAC). Mice were given a two-dose vaccination of parental BCG, rBCG1, or rBCG2 (5 × 10^5^ colony forming units [CFUs] per mouse) subcutaneously at weeks 0 and 2. Mice were then challenged with 10^6^ CFU of H37Ra per mouse at week 4 by intravenous injection. For macrophage depletion, mice were given a two-dose vaccination of parental BCG, rBCG1, or rBCG2 (5 × 10^5^ CFU per mouse) subcutaneously at weeks 0 and 2, and they received one drop of encapsome® (control) or clodrosome® (Encapsula Nano Sciences, TN, USA) intratracheally weekly for 8 weeks. For the biosafety assay, C57BL/6 and SCID mice aged 6–8 weeks were given a two-dose vaccination of parental BCG, rBCG1, or rBCG2 (5 × 10^5^ CFU per mouse) subcutaneously at weeks 0 and 2, and then challenged with H37Ra at week 4. The mice were closely monitored and their body weights were measured every week until week 40 (or a humane endpoint).

### Multiple Cytokine Testing

After sacrifice, blood was collected and plasma was stored at −20°C until use. Murine inflammatory cytokine testing was performed using a multiple mouse cytometric bead array (CBA) mouse inflammation kit (BD Biosciences, San Jose, CA, USA).

### Immunohistochemical Staining (IHC) and Pathologic Score

Samples of lung tissue were fixed in formalin and embedded in paraffin using routine methods, and the sections were then stained with hematoxylin and eosin (H&E). Tissue processing was performed by the core pathology facility at the National Health Research Institutes (Maoli, Taiwan). Histopathologic parameters (i.e., peribronchiolitis, perivasculitis, alveolitis, and granuloma formation) were each semi-quantitatively scored as absent, minimal, slight, moderate, marked, or strong using numerical scores of 0, 1, 2, 3, 4, and 5, respectively (28). Lesion frequency and severity were incorporated into these scores. For each time point, lung tissue from six or seven animals was examined, and the mean score for each slide of five or six random views was calculated. For IHC, paraffin sections were rehydrated and retrieved with 100 mM citrate buffer (pH 6.0) at 100°C for 5 min. After blocking the peroxidase activity and background (IHC/ICC kit, BioVision, Milpitas, CA, USA), the serial sections were incubated with primary antibody anti-iNOS (Abcam, ab15323, Cambridge, UK, **RRID:AB_301857**) and anti-mannose receptor (Abcam, ab64693, Cambridge, UK, **RRID:AB_1523910**), and stained following the manufacturer's protocol (BioVision). Finally, the sections were colored with the chromogen DAB and counterstained with hematoxylin. For determination of positive cells, the number of DAB-positive plus hematoxylin- positive cells in six randomly selected fields per slide (each an area of 8.4 × 10^3^ μm^2^) was calculated and analyzed.

### Bacterial Culture

Half of each lung tissue sample was minced and passed through a MACS C tube to produce single-cell suspensions in 5 ml of saline (cell survival rate >99.5% by hemocytometer). CFU values were determined by using 100 μl of cell mixture dilution on 7H10 agar plates. Each plate contained 100 μl of tissue homogenate, and each sample was titrated for three dilutions (10×, 100× and 1000×) performed in triplicate. Plates were kept at 37°C for 3–4 weeks. The colony number was counted and presented as a value per one lung (one mouse).

### Flow Cytometry

Lung and spleen tissues were minced and passed through a MACS C tube to produce single-cell suspensions (cell survival rate >99.5% by hemocytometer). The cells were incubated in 50 μl of RPMI medium that contained 2% fetal bovine serum (FBS) with Fc receptor for 15 min and then with several antibodies: CD4-FITC (cat. 553046, **RRID:AB_394582**), CD4-PerCP-Cy5.5(cat. 553052, **RRID:AB_394587**), CD8-PerCP-Cy5.5 (cat. 551162, **RRID:AB_394081**), CD25-BB515(cat.564424, **RRID:AB_2738803**), F4/80-PE(cat. 565410, **RRID:AB_2687527**), CD49b-APC, (cat.558295, **RRID:AB_398658**), IFN-γ-FITC(cat.554411), IL-17A-AF647(560184, **RRID:AB_1645204**), IL-4-PE(cat. 554435, **RRID:AB_395391**), Foxp3-PE(cat. 560408, **RRID:AB_1645251**) (all from BD Biosciences), and CD317-PerCP-Cy5.5 (pDCs; 120G8, Dendritics, San Diego, CA, USA, **RRID:AB_2566646**). For intracellular cytokine labeling, cells were stimulated with MTB-specific peptides (5 μg/ml of CD4 peptide Ag85B_241−255aa_ QDAYNAAGGHAVFN (Mission Biotech, Taiwan, cat. 991172), CD8 peptide Ag85B_1−19aa_ FSRPGLPVEYLQVPSMG, Mission Biotech, Taiwan, cat. 991171) for 68 h and then Golgi-stop (BD Biosciences) was added for an additional 4 h [28). Cells were then fixed, permeabilized and stained with cytokine antibodies (IFN-γ-FITC, IL-17A-AF647, IL-4-PE and Foxp3-PE) for 1 h following standard procedures. The gating strategy involved CD4^+^ T cells, and other relevant cell populations were gated from the single cell lymphocyte population in the forward scatter (FSC) and side scatter (SSC) sections (29). The samples were acquired and analyzed using a FACSCalibur system and CellQuest software (BD Biosciences).

### RNA Extraction and Quantitative PCR

Total mRNA was extracted by Trizol reagent, and cDNA was prepared by using a reverse transcription kit (ThermoFisher Scientific, CA, USA). Primer sequences are shown in Table 1. The mRNA levels of TNF-α, IFN-γ, MCP-1, various transcription factors and GAPDH were detected by real-time quantitative PCR analysis using the ABI vii7 system (Applied Biosystems, Foster City, CA, USA). Cytokine and GAPDH levels were calculated relative to amounts found in a standard sample, and cytokine levels were corrected for GAPDH mRNA levels to normalize for RNA input. Relative expression (Relative index) is presented as 2^−ΔCT^.

**Table 1:** Sequences of primers used for quantitative PCR.

**Primer name**	**Sequence (5'–3')**
mTNF-α	Forward	AAGCCTGTAGCCCACGTCGTA
	Reverse	GGCACCACTAGTTGGTTGTCTTTG
mIFN-γ	Forward	CATTGAAAGCCTAGAAAGTCTGAATAAC
	Reverse	TGGCTCTGCAGGATTTTCATG
mIL-4	Forward	CCTCACAGCAACGAAGAACA
	Reverse	TGGACTCATTCATGGTGCAG
mIL-5	Forward	CGCTCTTCCTTTGCTGAAG
	Reverse	TAGGGACAGGAAGCCTCATC
mIL-6	Forward	AAGTCGGAGGCTTAATTACACATGT
	Reverse	AAGTGCATCATCGTTGTTCATACA
mIL-10	Forward	GCTCTTACTGACTGGCATGAG
	Reverse	CGCAGCTCTAGGAGCATGTG
mIL-12	Forward	GGAAGCACGGCAGCAGAATA
	Reverse	AACTTGAGGGAGAAGTAGGAATGG
mIL-17	Forward	ATCCACCTCACACGAGGCAC
	Reverse	ACCTTCACATTCTGGAGGAA
mIFN-α1	Forward	AGTGAGCTGACCCAGCAGAT
	Reverse	CAGGGGCTGTGTTTCTTCTC
mIFN-β1	Forward	CCCTATGGAGATGACGGAGA
	Reverse	CTGTCTGCTGGTGGAGTTCA
miNOS	Forward	AAAGTGACCTGAAAGAGGAAAAGGA
	Reverse	TTGGTGACTCTTAGGGTCATCTTGTA
mGAPDH	Forward	GTTGTCTCCTGCGACTTCA
	Reverse	GGTGGTCCAGGGTTTCTTA
mIDO1	Forward	TGGCACTCAGTAAAATATCTCCT
	Reverse	CAGGCAGATTTCTAGCCACA
mIDO2	Forward	ATGGAGCCTCAAAGTCAGAGC
	Reverse	CGCTGCTCACGGTAACTCTTTA
mMCP-1	Forward	CCCACTCACCTGCTGCTACT
	Reverse	TCTGGACCCATTCCTTCTTG

### Statistical Analysis

All results are presented as mean ± SEM for six or seven mice per group. The statistical significance between the experimental groups was assessed using a two-tailed unpaired Student's *t*-test, non-parametric analysis, one-way and two-way analysis of variance (ANOVA) with a Tukey or Bonferroni *post hoc* test. The differences were considered significant for *P* < 0.05. Statistical tests were performed using GraphPad Prism version 6.0 (GraphPad Software, La Jolla, CA, USA).

## Results

### rBCG1 and rBCG2 Vaccines Protect Mice Against *Mycobacterium tuberculosis* H37Ra

**Figure 1:**
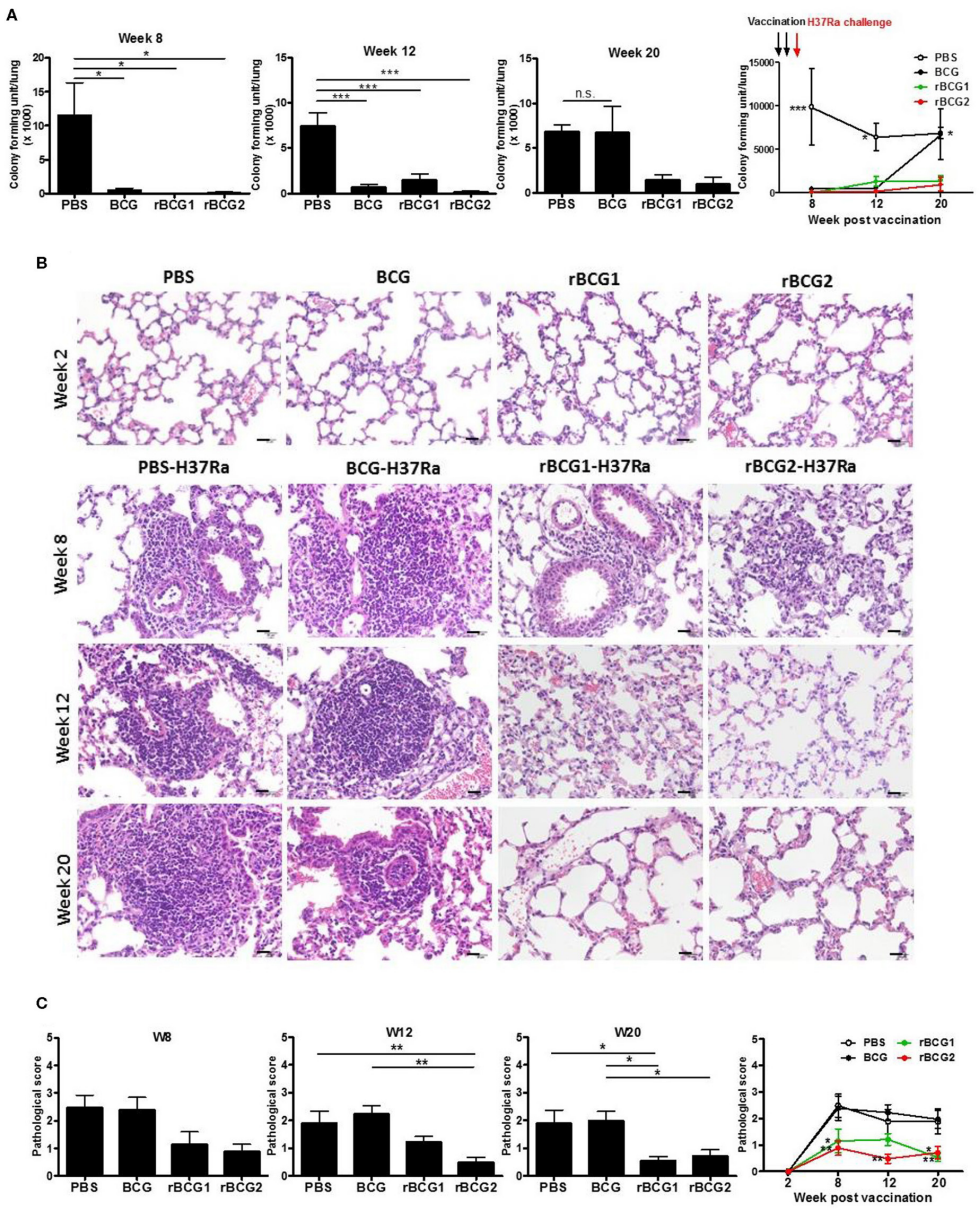
Protection provided by recombinant BCG vaccines rBCG1 and rBCG2 against *Mycobacterium tuberculosis* H37Ra in mice. C57BL/6 mice were immunized with BCG, rBCG1, or rBCG2 at weeks 0 and 2, and then challenged with H37Ra at week 4 (*n* = 6–7, in two independent experiments). At 2, 8, 12, and 20 weeks, the mice were sacrificed. One lung from each mouse was homogenized and the other was fixed in 3.7% formaldehyde. **(A)** Colony forming units (CFU) of bacterial growth in murine lung. Each plate contained 100 μl of tissue homogenate, and each sample was titrated for two dilutions (5× and 50×) and repeated in triplicate. **(B)** Lung samples were fixed in 3.7% formaldehyde, embedded in paraffin, sectioned, and stained with H&E; scale bar = 20 μm. Differences among groups were determined using a one-way, non-parametric test, or two-way ANOVA with a Tukey or Bonferroni *post hoc* test (*P < 0.05; **P < 0.01; ***P < 0.001; ns, non-significant). **(C)** Pathologic scores were determined and analyzed by H&E staining. For each time point, lung tissue from six or seven animals was examined and the mean score for each slide of five or six random views was calculated. Differences among groups were determined by one-way or two-way ANOVA with a Tukey or Bonferroni *post hoc* test (*P < 0.05, **P < 0.01).

To determine whether our recombinant BCG vaccines rBCG1 and rBCG2 protect against MTB infection, we first conducted a vaccine efficacy test against the H37Ra strain in an ABSL2 facility. (We would have conducted this initial test in an ABSL3 facility with an aerosol virulent MTB Beijing strain, however our facility was not operational at the time.) We administered the vaccines to mice subcutaneously twice (at weeks 0 and 2) and then challenged the mice with MTB H37Ra via intravenous injection at week 4. The bacterial load and lung pathology were measured at weeks 8, 12, and 20 after the immunization. At week 2 (prior to H37Ra challenge), the lungs showed no sign of cellular infiltration (Figures 1B,C). At week 8 post-vaccination, the bacterial load of the PBS control group was high [CFU = (115.2 ± 47.91) × 1,000] compared to that of the BCG [CFU = (5.0 ± 2.822) × 1,000], rBCG1 [CFU = (0 ± 0) × 1,000] and rBCG2 [CFU = (1.112 ± 1.112) × 1,000] groups (^**^P < 0.001; Figure 1A). Inflammation in the PBS control group was more severe than in the vaccination groups (Figures 1B,C). At week 12, the bacterial load in the PBS control was still high [CFU = (74.72 ± 14.13) × 1,000], whereas the bacterial loads were lower in the BCG, rBCG1, and rBCG2 groups [CFU = (6.66 ± 2.974) × 1,000, (14.99 ± 5.867) × 1,000, and (1.667 ± 1.054) × 1,000, respectively, ^**^P < 0.001; Figure 1A]. However, the cellular infiltration lesions had nearly disappeared by week 12 in the rBCG1 and rBCG2 groups (Figures 1B,C). Thus, the rBCG1 and rBCG2 vaccines protected mice against H37Ra infection at an early stage. At week 20, stable bacterial loads were detected for the PBS control group [CFU = (68.57 ± 6.904) × 1,000], but very low bacterial loads were measured in the rBCG1 and rBCG2 groups [CFU = (16.67 ± 5.236) × 1,000 and (11.11 ± 9.165) × 1,000, respectively; ^*^P < 0.05]. At 20 weeks, the bacterial load had dramatically increased in the BCG group [CFU = (66.9 ± 29.23) × 1,000, *P* > 0.05; Figure 1A). These CFU results paralleled the H&E staining patterns and pathologic scores (Figures 1B,C). Taken together, these results suggest that, whereas the parental BCG vaccine does not provide adequate protection against H37Ra infection, the rBCG1 and rBCG2 vaccines do. Thus, the efficacies of the rBCG1 and rBCG2 vaccines are higher than that of the parental BCG.

### rBCG1 and rBCG2 Vaccines Are Safe in SCID Mice as Well as in C57BL/6 Wild-Type Mice

**Figure 2:**
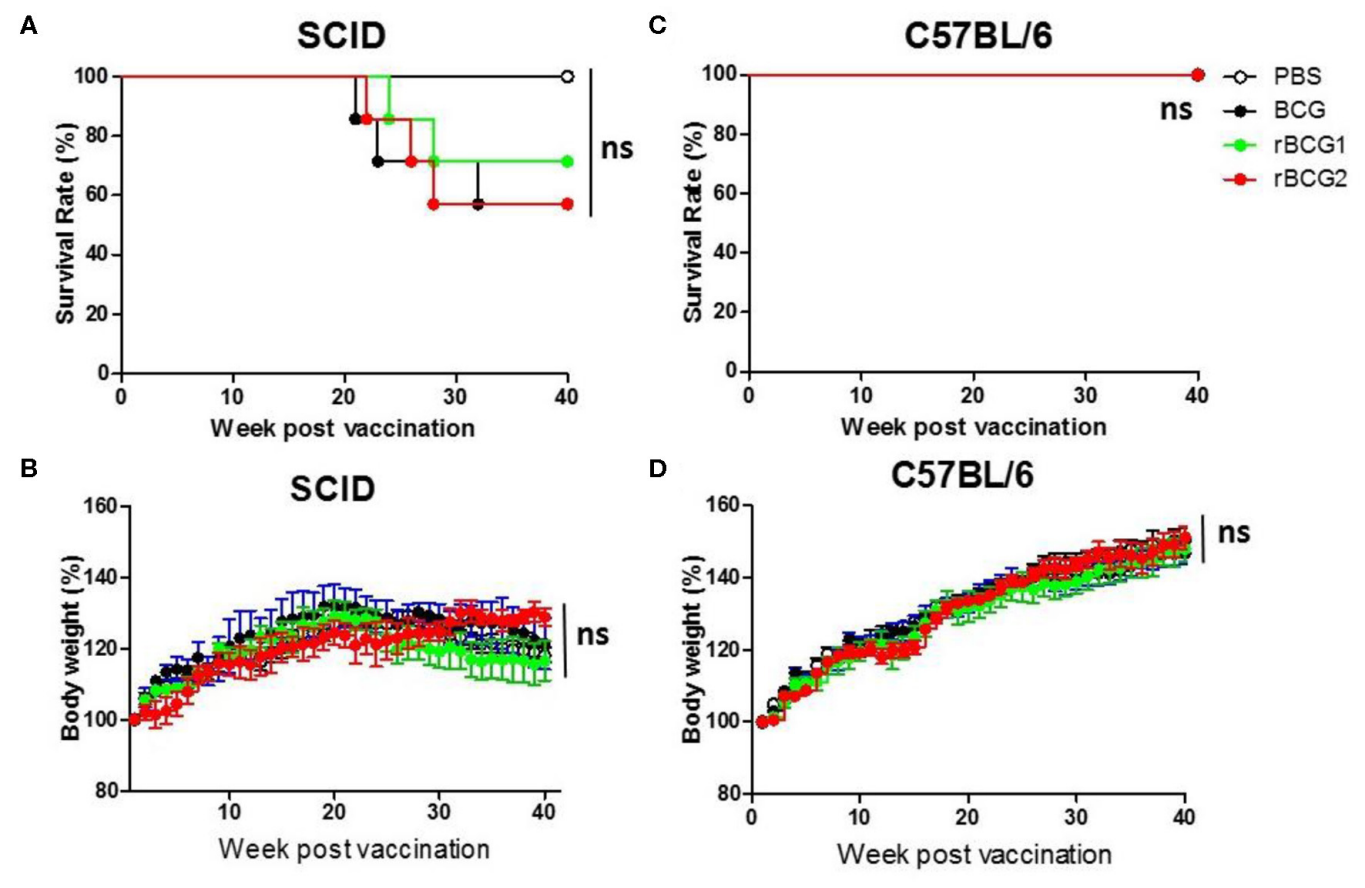
Test of rBCG vaccine safety in immunodeficient and immunocompetent mice. SCID and C57BL/6 wild-type mice were immunized with BCG, rBCG1, or rBCG2 at weeks 0 and 2 (*n* = 10). **(A,C)** Survival rate of SCID and C57BL/6 mice after vaccination. **(B,D)** The body weights of SCID and C57BL/6 mice were recorded every week. Body weight (%) was calculated as: weight (week n)/weight (week 0) per mouse. Survival curves were plotted by the Kaplan-Meier method. The Log-rank test was performed to compare each pair of survival curves (*P* > 0.05; ns, non-significant). Differences among groups were determined by two-way ANOVA with a Bonferroni *post hoc* test (*P* > 0.05; ns, non-significant).

To test the safety of the rBCG1 and rBCG2 vaccines, we gave the same dose of BCG and rBCG vaccines to severe combined immunodeficiency (SCID) and C57BL/6 wild-type mice subcutaneously at weeks 0 and 2. All of the mice were monitored very closely until week 40 or an earlier humane endpoint. Mouse weight was recorded every week. After the vaccination, neither the SCID nor wild-type mice showed any adverse physical signs. The weights of both groups increased slightly every week, but the differences between groups were not significant (Figures 2B,D, *P* > 0.05, ns). Starting at week 21, one SCID mouse in each of the BCG, rBCG1 and rBCG2 groups was euthanized for humane reasons but there were no deaths in the PBS group (Figure 2A) or in any of the wild-type groups (Figure 2C). These results suggest that the rBCG1 and rBCG2 vaccines are safe, even in immunodeficient individuals.

### mRNA Expression of Th1-Mediated Cytokines in Mouse Lung Tissue Following Immunization With BCG, rBCG1, and rBCG2

**Figure 3:**
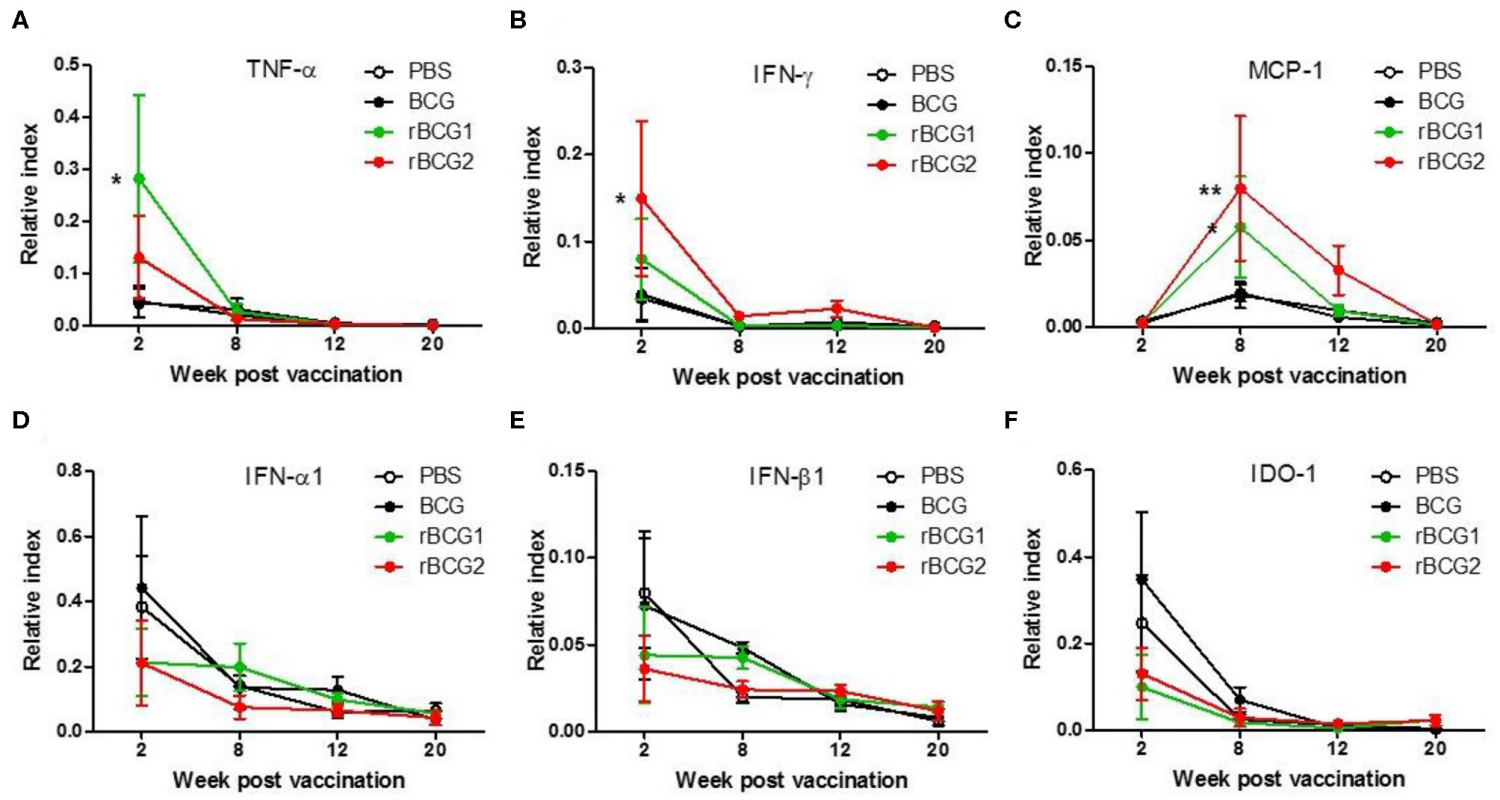
Th1-mediated cytokine profiles expressed in murine lung following immunization with rBCG1 or rBCG2. C57BL/6 mice were immunized with BCG, rBCG1 or rBCG2 at weeks 0 and 2. They were then challenged with H37Ra at week 4 (*n* = 6–7, in two independent experiments). At 2, 8, 12, and 20 weeks, the mice were sacrificed, lung RNA was extracted and cytokine gene expression profiles were determined using qPCR. **(A)** TNF-α, **(B)** IFN-γ, **(C)** MCP-1, **(D)** IFN-α1, **(E)** IFN-β1, **(F)** IDO-1. Differences among groups were determined using two-way ANOVA with a Bonferroni *post hoc* test (*P < 0.05; **P < 0.01).

We next examined cytokine expression in the lungs using quantitative PCR (qPCR) to determine whether recombinant BCG vaccines stimulate Th1-mediated cytokine production. RNA was extracted from cells isolated from the lungs and spleens of the PBS control and vaccination groups before and after H37Ra challenge, at weeks 2, 8, 12, and 20, and qPCR was performed to detect cytokine gene expression in these tissues. TNF-α, IFN-γ, and MCP-1 were all increased in the BCG, rBCG1 and rBCG2 groups compared to the PBS control group, particularly during weeks 2 and 8 (Figures 3A–C). At weeks 12 and 20, TNF-α, IFN-γ, and MCP-1 were decreased in all groups (Figures 3A–C); presumably this reflects bacterial clearance, when cytokine production stops and normal resting levels are reached. These cytokines are the products of Th1-mediated immune responses. In contrast, IFN-α1, IFN-β1, and IDO-1, which are secreted by some of the surrounding infected immune cells, were detected at higher levels in the PBS and BCG groups compared to the rBCG1 and rBCG2 groups (Figures 3D–F). Cytokines IL-1β, IL-4, IL-6, IL-10, IL-12, IL-17, and IDO-2 were unchanged or undetectable for all groups (data not shown). These results suggest that rBCG1 and rBCG2 can induce host Th1 responses to clear MTB bacteria.

### Macrophages, Th1 Cells and Th17 Cells Dominate Following Immunization With BCG, rBCG1, and rBCG2

**Figure 4:**
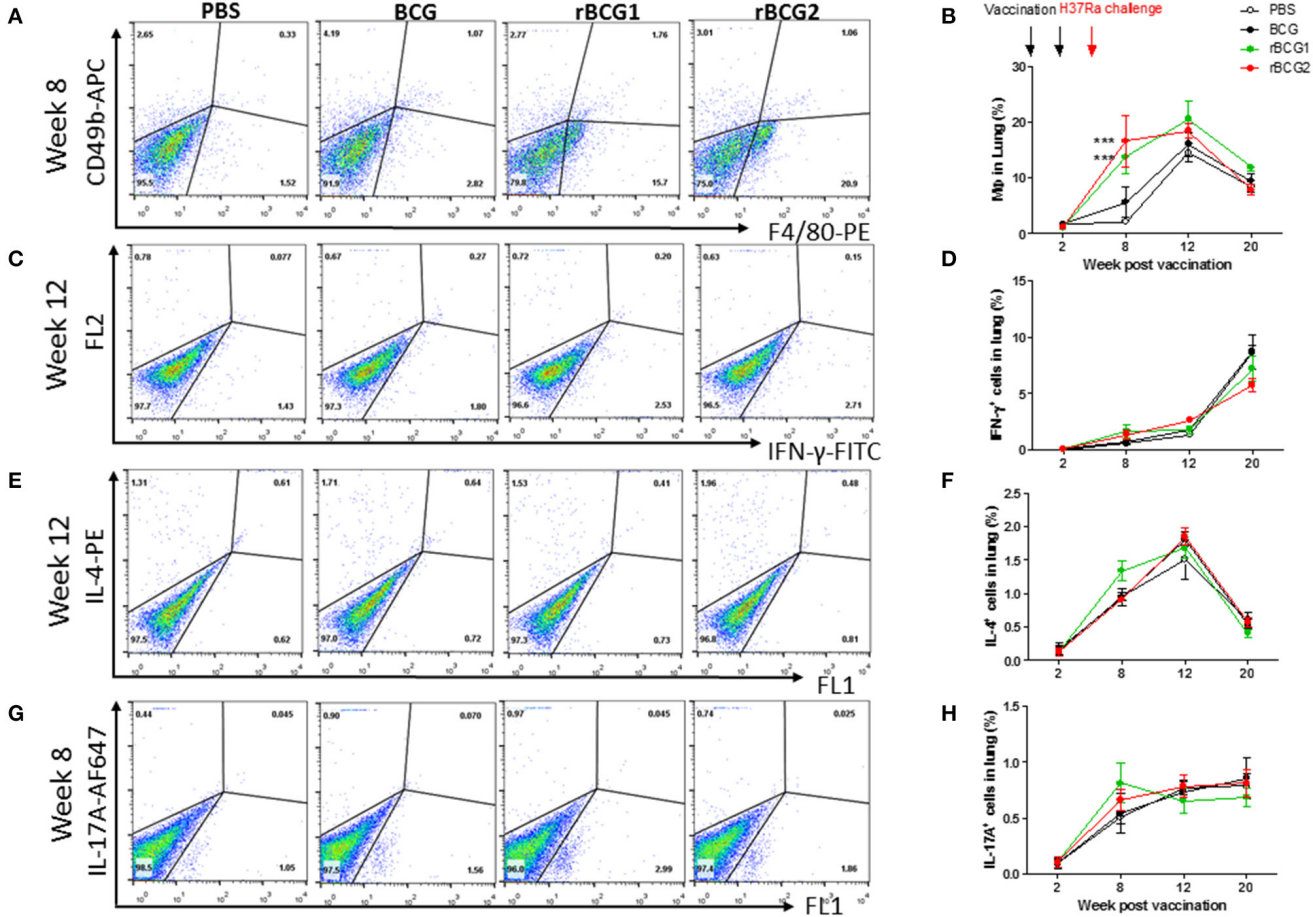
Innate and adaptive immune cell profiles pulsed with tuberculosis-specific peptides from mice immunized with BCG, rBCG1, or rBCG2. C57BL/6 mice were immunized with BCG, rBCG1 or rBCG2 at weeks 0 and 2, and then challenged with H37Ra at week 4 (*n* = 6–7, in two independent experiments). At 2, 8, 12, and 20 weeks, the mice were sacrificed, and lungs were homogenized to single-cell suspensions. The cells were stimulated with tuberculosis-specific TB peptides (described in the Materials and Methods) for 68 h and then Golgi-stop for 4 h, stained for surface and intracellular markers, and then subjected to flow cytometry to determine the percentage of cytokine-producing cells within CD4^+^ T cells. **(A)** Percentage of macrophages (F4/80^+^ cells) in lung post-vaccination at week 8. **(B)** Percentage of macrophages (F4/80^+^ cells) in lung post-vaccination at weeks 2–20. **(C)** Percentage of IFN-γ^+^ cells in lung post-vaccination at week 12. **(D)** Percentage of IFN-γ^+^ cells in lung post-vaccination at weeks 2–20. **(E)** Percentage of IL-4^+^ cells in lung post-vaccination at week 12. **(F)** Percentage of IL-4^+^ cells in lung post-vaccination at weeks 2–20. **(G)** Percentage of IL-17^+^ T cells in lung post-vaccination at week 8. **(H)** Percentage of IL-17^+^ T cells in lung post-vaccination at weeks 2–20. Differences among groups were determined by one-way or two-way ANOVA with a Tukey or Bonferroni *post hoc* test (***P < 0.001).

We analyzed immune cell profiles from murine lung and spleen tissue using flow cytometry. Single cells were isolated from the lung and spleen in the PBS control and vaccination groups before and after H37Ra challenge, at weeks 2, 8, 12, and 20. After conducting surface marker and intracellular staining, FACS was used to analyze immune cell profiles. At week 2, all immune cell levels were very low, with no differences among the four groups (Figures 4B,D,F,H). At week 8 (i.e., 4 weeks after H37Ra challenge), macrophage numbers (F4/80^+^ cells) had increased dramatically in the rBCG1 and rBCG2 vaccination groups compared to the PBS control and BCG groups (Figures 4A,B; ^*^P < 0.05). There were few NK cells (CD49b^+^ cells) and resident macrophages in all groups at 2 weeks (data not shown). Increased IFN-γ^+^ T cells (Figures 4C,D) and IL-17A^+^ T cells (Figures 4G,H) (but not IL-4^+^ T cells, Figures 4E,F) were also detected in all BCG vaccination groups, but especially in the rBCG1 and rBCG2 groups. Thus, the induced cellular immunity was higher in mice immunized with rBCG1 and rBCG2 compared to parental BCG.

### Monocyte Chemoattractant Protein-1 (MCP-1) Is the Dominant Cytokine Produced in Serum Following rBCG1 and rBCG2 Vaccination

**Figure 5:**
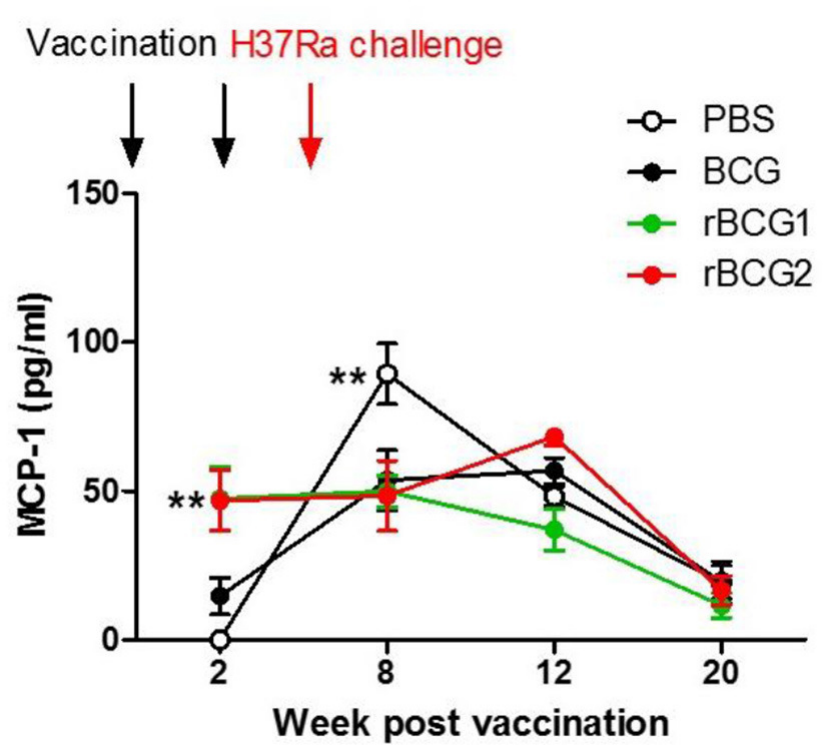
High MCP-1 expression in serum during early-stage recombinant BCG vaccination. C57BL/6 mice were immunized with BCG, rBCG1, and rBCG2 at weeks 0 and 2, and then challenged with H37Ra at week 4 (*n* = 6–7, in two independent experiments). At 2, 8, 12, and 20 weeks, sera were collected, diluted 20,000 times, and assayed using a multiple cytokine kit. Differences among groups were determined by two-way ANOVA with a Bonferroni *post hoc* test (**P < 0.01).

Cytokine production was measured for serum and tissue (lung and spleen) using cultured supernatant samples and a multiple cytokine inflammation kit (BD Biosciences). The cytokine detection kit measured all inflammatory cytokines, including TNF-α, IFN-γ, IL-6, IL-10, IL-12, and MCP-1. No cytokines were found in tissue culture supernatant samples (data not shown) as they were below the limit of detection. Interestingly, MCP-1 was the only cytokine detected in serum. MCP-1 increased in the serum of mice immunized with rBCG1 and rBCG2 at 2 and 8 weeks (Figure 5; ^**^P < 0.01). MCP-1 also increased in the PBS control and BCG groups beginning at week 8 and continuing until week 12. MCP-1 then decreased in all groups at week 20. The high MCP-1 production (weeks 2 and 8) coincided with reduced bacterial loads (Figure 1A) and increased lung macrophage levels in the rBCG1 and rBCG2 groups (Figure 4A).

### Macrophages Are Key Mediators of the Immune Response at the Early Stage of H37Ra Infection

**Figure 6:**
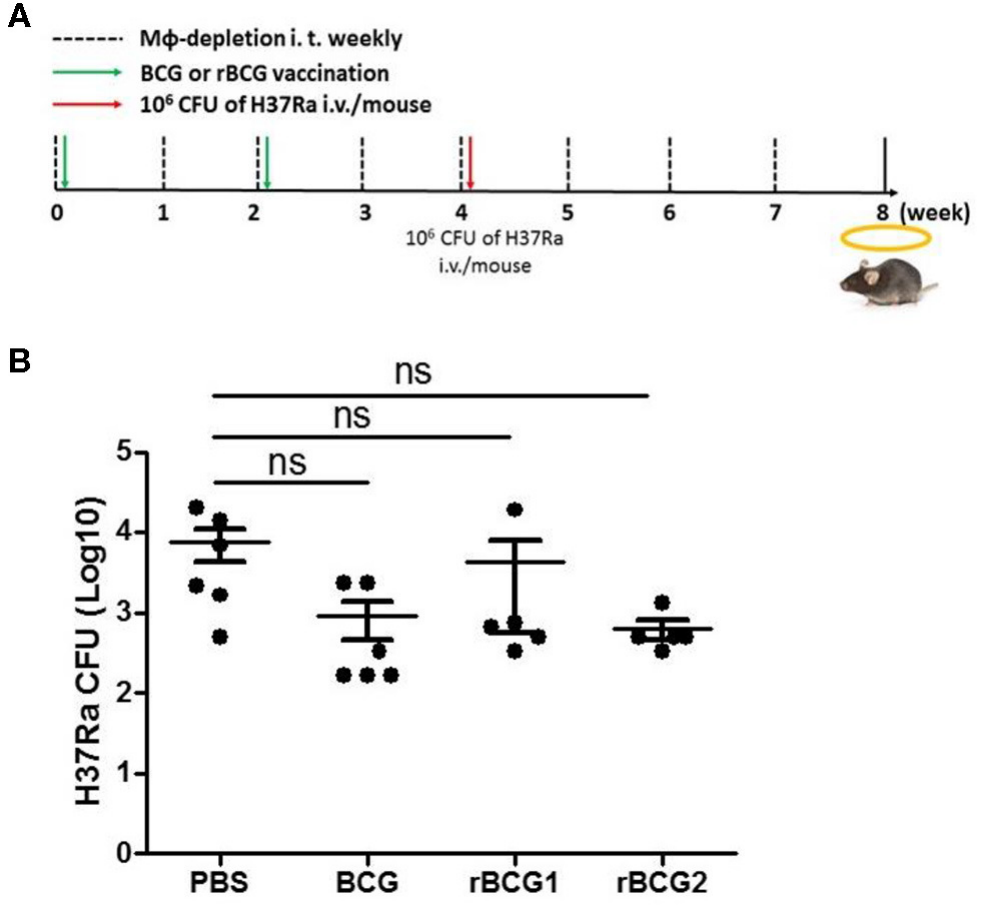
Macrophages are significant for protection against *Mycobacterium* infection. C57BL/6 mice were given a two-dose vaccination of parental BCG, rBCG1, and rBCG2 (5 × 10^5^ CFU per mouse) subcutaneously at weeks 0 and 2 (*n* = 6), and a challenge of H37Ra at week 4; they also received 50 μl of encapsome® (control) or clodrosome® intratracheally weekly for 8 weeks to deplete macrophages. Mice were sacrificed at week 8. **(A)** Timeline for vaccination, challenge, and intratracheal inoculation in mice. **(B)** CFU of bacterial growth in murine lung at week 8. Each plate contained 100 μl of tissue homogenate, and each sample was titrated for two dilutions (5× and 50×) and repeated in triplicate. Differences among groups were determined using one-way ANOVA with a Tukey *post hoc* test (*P* > 0.05, ns, non-significant).

Based on the qPCR results, the increased MCP-1 production, and the higher numbers of macrophages in the lungs, macrophage function is obviously of central importance in the response to MTB infection. To evaluate the role of macrophages early in infection, we attempted to deplete macrophages by administering clodronate-liposomes intratracheally every week following the vaccination and challenge (Figure 6A). After 3–4 weeks, we counted and calculated the bacterial load among the control and vaccination groups. The bacterial load of the PBS control group was still high [CFU = (7.611 ± 3.282) × 1,000] (Figure 6B); however, the bacterial loads of each of the vaccination groups (BCG [CFU = (0.9167 ± 0.4488) × 1,000], rBCG1 [CFU = (4.283 ± 3.722) × 1,000], rBCG2 [CFU = (0.6333± 0.178) × 1,000]) were dramatically increased (compared with the results shown in Figure 1A), but were not significantly different compared to the PBS group (Figure 6B, *P* > 0.05, ns). This result suggests that macrophages are the main immune cells involved in protection against *Mycobacterium* infection early on. After macrophage depletion, the mice were no longer protected against the infection.

### Increased Levels of Inflammatory M1-Polarized Macrophages Especially in rBCG1 and rBCG2 Vaccinated Mice

**Figure 7:**
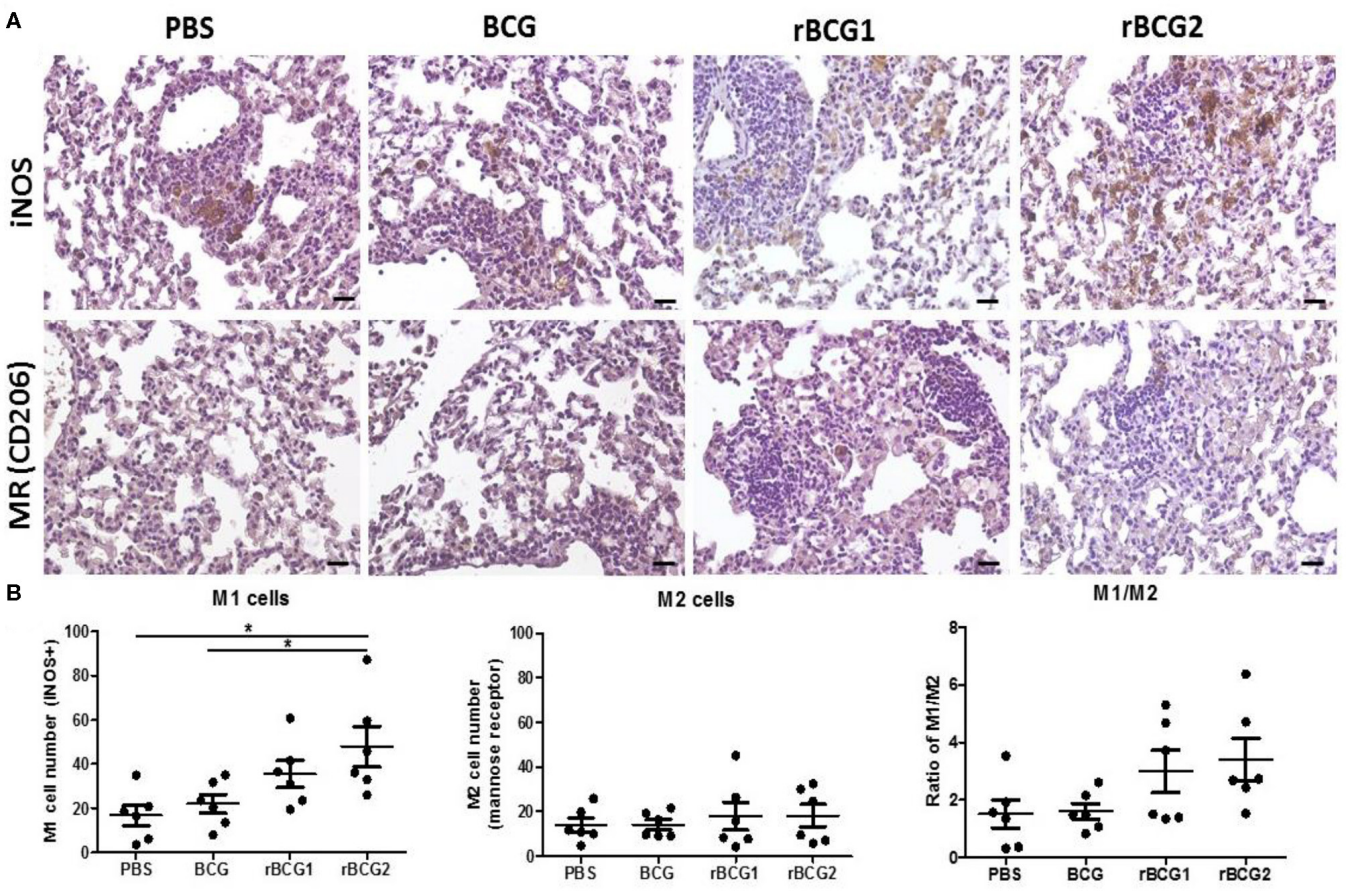
Increased M1-polarized macrophage cells in lung tissue sections from mice vaccinated with rBCG1 or rBCG2. C57BL/6 mice were immunized with BCG, rBCG1, or rBCG2 at weeks 0 and 2, and then challenged with H37Ra at week 4 (*n* = 6–7). At week 8, the mice were sacrificed. One lung from each mouse was homogenized and the other lung was fixed in 3.7% formaldehyde. **(A)** After fixation, lung samples were embedded in paraffin, sectioned, and stained for iNOS (M1 marker) and MR (M2 marker); scale bar = 20 μm. The images are representative of slides from the four groups. **(B)** Lung tissue from six or seven animals per group was examined, and the number of DAB^+^ plus hematoxylin-positive cells for each slide, based on five or six random views (each field of view has an area of 8.4 × 10^3^ μm^2^), was calculated. Differences among groups were determined by one-way ANOVA with a Tukey *post hoc* test (*P < 0.05).

Macrophages adapt to the microenvironment and differentiate to express different functional phenotypes. M1 macrophages produce high levels of pro-inflammatory cytokines and have a strong ability to kill pathogens. In contrast, M2 macrophages are involved in clearance of parasites, tissue remodeling and immune regulation (30). We believe that rBCG1 and rBCG2 enhance the ability of M1 macrophages to clear pathogens. Using the remaining tissue samples fixed in paraffin blocks, we sought to compare the levels of M1 macrophages in the vaccination groups, especially in the rBCG1 and rBCG2 groups. We examined paraffin sections collected at week 8 because they showed the highest macrophage numbers and MCP-1 production. After deparaffinization, rehydration, antigen retrieval and blocking, the serial sections were stained to measure inducible nitric oxide synthase (iNOS, M1 macrophage marker) and mannose receptor (MR, M2 macrophage marker). In general, each treatment group expressed different levels of iNOS (Figure 7A); however, iNOS-expression was raised dramatically in the rBCG1 and rBCG2 groups (the mean M1 number for rBCG1 was ~35, for rBCG2 ~50, compared to PBS ~18 and BCG ~22; Figures 7A,B, ^*^P < 0.05). The expression of MR was similar in all groups (the mean M2 value was ~15–20, Figures 7A,B). These results indicate that, among the tested vaccines, rBCG2 induces the greatest increase in the number of M1 macrophages to clear bacteria in the lungs.

## Discussion

Ag85B and CFP-10 are well-known MTB antigens that strongly induce IFN-γ production (31) and enhance T cell proliferation (31, 32). Both proteins are considered good vaccine candidates for tuberculosis. Notably, IL-12 is believed to play an important role in cell-mediated immunity against intracellular infection, primarily by acting on T and NK cells. Recent study findings reveal that IL-12 can activate macrophages potently during intracellular infection, and this activating effect is mediated primarily through its effect on macrophage IFN-γ release (33). Therefore, rBCG2 expressing *Mycobacterium*-specific Ag85B and CFP10 combined with human IL-12 was constructed and investigated in this study. In our previous study, protection efficacy was tested by using splenocytes from vaccine-immunized mice and co-culturing them with *ex vivo* murine bone-marrow-derived macrophages to measure the survival ability of the MTB strains (34). The results showed that rBCG2 induced a strong immune response against MTB infection compared with the parental BCG strain (34). In the present study, we first tested rBCG1 and rBCG2 against the MTB strain H37Ra. We found that rBCG1 and rBCG2 produced a stronger immune response to MTB infection compared to the parental BCG vaccine in mice. Previous *ex vivo* murine bone-marrow-derived macrophage infection experiments with H37Rv also showed that rBCG1 and rBCG2 provide stronger immunity compared to parental BCG (35, 36). In the present study, during early infection, all three tested vaccines (BCG, rBCG1 and rBCG2) inhibited H37Ra growth. However, at week 20, the bacterial load in the BCG group rebounded and increased. In contrast, the bacterial loads in the rBCG1 and rBCG2 groups remained low and nearly undetectable. If the murine age is converted to human age, the period of protection of BCG (20 weeks) found in this study would be equivalent to 15–20 years; this finding is consistent with that of a previous epidemiologic study of BCG vaccination in humans (37). Thus, rBCG1 and rBCG2 could provide more effective and longer protection against H37Ra compared to parental BCG.

We showed that macrophages, IFN-γ^+^ cells and IL-17^+^ cells increase soon after vaccination, particularly following rBCG1 and rBCG2 vaccination. Detection of high-level RNA expression of TNF-α, IFN-γ and MCP-1 in lung tissue of mice immunized with rBCG1 and rBCG2 further supports the immuno-protective results for these two candidate vaccines. MCP-1 produced in response to infection recruits monocytes, dendritic cells and T cells to the site of inflammation caused by infection. More importantly, in the present study, MCP-1 was the only cytokine detectable in mouse sera in the early stages following rBCG1 and rBCG2 vaccination. Although MCP-1 expression also increased in the PBS control group at 8 weeks, this finding is expected in the natural course of infection. These results also fit with our finding of increased macrophages in lung tissue following rBCG1 and rBCG2 vaccination. Thus, macrophages are vital to the early host immune response following rBCG1 and rBCG2 vaccination, but not parental BCG vaccination. Furthermore, the bacterial loads in the vaccination groups (BCG, rBCG1, and rBCG2) depleted of alveolar macrophages rose significantly, illustrating the importance of the protective role of macrophages early in MTB infection, which has not been shown previously. Moreover, we also found more inflammatory M1 cells in the two rBCG groups compared to the PBS control and parental BCG groups. This latter finding supports our argument that macrophages play a more important role in early and adaptive immunity to H37Ra than previously believed. Functional M1 macrophage assays will be needed to further clarify their roles. Over the past decades, most efforts to develop TB vaccine candidates have focused on enhancing adaptive immunity. However, in phase 2b clinical trials, while these vaccines have induced strong immune responses, they have been unable to confer significant protection against TB infection (38, 39). Recent studies suggest that trained immunity, particularly involving macrophages, may play a more important role than T cells in vaccine-mediated immunity (40–44), creating a new prospective view for vaccine development. Notably, our results suggest that both innate and adaptive immunity play important roles against *Mycobacterium* infection.

This study had some limitations. First, because our ABSL3 facility was not operational during the initial study period, we first conducted a vaccine efficacy test against the H37Ra strain in an ABSL2 facility. The present study not only focused on pathogenesis but also on the protective effect of vaccines against H37Ra, which is often used in vaccine or drug efficacy evaluation (45–47). To evaluate the efficacy of the new recombinant BCG, both *in vivo* (H37Ra infection, this study) and *ex vivo* models [H37Rv infection (34)] have been performed in our laboratory. Our results suggest that rBCG vaccines can provide good, safe and long-term protection against H37Ra infection. Second, functional M1 macrophage assays will be needed to further clarify the roles of macrophages. Our results showed that rBCG vaccination stimulated the greatest increase in the number of M1 macrophages, presumably to clear bacteria in the lungs, which may be involved in long-term protection.

In summary, this study showed that our rBCG vaccines are safe in SCID mice, and that they induced a significant increase in the number of M1 macrophages. Moreover, to further verify vaccine efficacy, an animal model incorporating a protective assay and challenged with virulent MTB strains should be investigated forthwith. Our results suggest that rBCG1 and rBCG2 may be good candidates as a novel TB vaccine.

## Data Availability Statement

All datasets generated for this study are included in the article/supplementary material.

## Ethics Statement

The animal study was reviewed and approved by National Health Research Institutes Institutional Animal Care and Use Committee (NHRI-IACUC).

## Author Contributions

S-JY designed, performed the experiments, analyzed the data and wrote the manuscript. C-HH, Y-YC, C-WH, C-YC, and J-RC performed and assisted in the experiments. H-YD supervised this work and interpreted the data. All authors contributed to the article and approved the submitted version.

## Conflict of Interest

The authors declare that the research was conducted in the absence of any commercial or financial relationships that could be construed as a potential conflict of interest.
