# Corrigendum: Activation of M1 Macrophages in Response to Recombinant TB Vaccines With Enhanced Antimycobacterial Activity

**DOI:** 10.3389/fimmu.2021.669616

**Published:** 2021-03-30

**Authors:** Shiu-Ju Yang, Yih-Yuan Chen, Chih-Hao Hsu, Chia-Wei Hsu, Chun-Yu Chang, Jia-Ru Chang, Horng-Yunn Dou

**Affiliations:** ^1^National Institute of Infectious Diseases and Vaccinology, National Health Research Institutes, Zhunan, Taiwan; ^2^Department of Biochemical Science and Technology, National Chiayi University, Chia-Yi, Taiwan

**Keywords:** recombinant Bacille Calmette–Guérin, *Mycobacterium tuberculosis*, innate immunity, macrophage, vaccine

In the original article, there was a mistake in **Figure 4G**. When analyzing the original data, the authors mistakenly took raw data of the second image of 4G, which caused the third and second images to be duplicated. The corrected **Figure 4** appears below.

**Figure 4:**
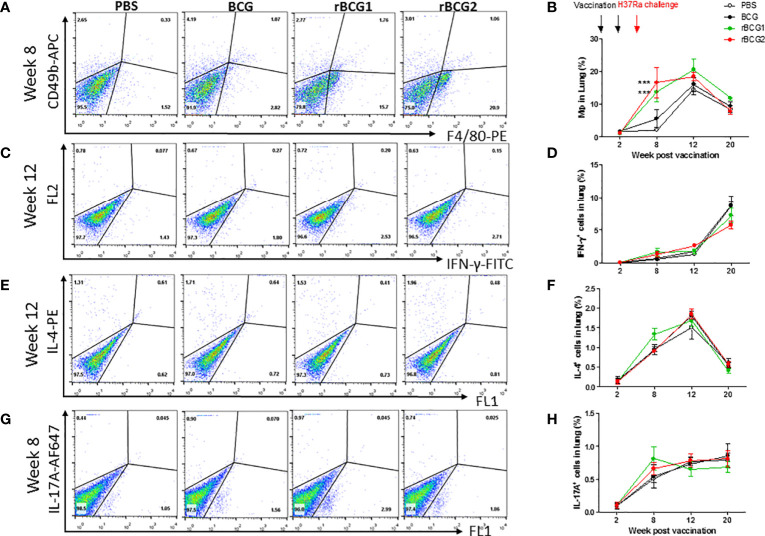
Innate and adaptive immune cell profiles pulsed with tuberculosis-specific peptides from mice immunized with BCG, rBCG1, or rBCG2. C57BL/6 mice were immunized with BCG, rBCG1 or rBCG2 at weeks 0 and 2, and then challenged with H37Ra at week 4 (n = 6 to 7, in two independent experiments). At 2, 8, 12, and 20 weeks, the mice were sacrificed, and lungs were homogenized to single-cell suspensions. The cells were stimulated with tuberculosis-specific TB peptides (described in the Materials and Methods) for 68 h and then Golgi-stop for 4 h, stained for surface and intracellular markers, and then subjected to flow cytometry to determine the percentage of cytokine-producing cells within CD4^+^ T cells. **(A)** Percentage of macrophages (F4/80^+^ cells) in lung post-vaccination at week 8. **(B)** Percentage of macrophages (F4/80^+^ cells) in lung post-vaccination at weeks 2 to 20. **(C)** Percentage of IFN-γ^+^ cells in lung post-vaccination at week 12. **(D)** Percentage of IFN-γ^+^ cells in lung post-vaccination at weeks 2 to 20. **(E)** Percentage of IL-4^+^ cells in lung post-vaccination at week 12. **(F)** Percentage of IL-4^+^ cells in lung post-vaccination at weeks 2 to 20. **(G)** Percentage of IL-17^+^ T cells in lung post-vaccination at week 8. **(H)** Percentage of IL-17^+^ T cells in lung post-vaccination at weeks 2 to 20. Differences among groups were determined by one-way or two-way ANOVA with a Tukey or Bonferroni *post hoc* test (***P < .001).

The authors apologize for this error and state that this does not change the scientific conclusions of the article in any way. The original article has been updated.

